# Prevalence and anatomical sites of human papillomavirus, Epstein-Barr virus and herpes simplex virus infections in men who have sex with men, Khon Kaen, Thailand

**DOI:** 10.1186/s12879-018-3406-0

**Published:** 2018-10-11

**Authors:** Jureeporn Chuerduangphui, Kanisara Proyrungroj, Chamsai Pientong, Saowarop Hinkan, Jiratha Budkaew, Charinya Pimson, Bandit Chumworathayi, Ploenpit Hanond, Tipaya Ekalaksananan

**Affiliations:** 1 Department of Microbiology, Faculty of Medicine, Khon Kaen University, Khon Kaen, Thailand; 2 Department of Social Medicine, Khon Kaen Center Hospital, Khon Kaen, Thailand; 3 Department of Animal Health Science, Faculty of Agro-Industrial Technology, Kalasin University, Kalasin, Thailand; 4 Department of Obstetrics and Gynecology, Faculty of Medicine, Khon Kaen University, Khon Kaen, Thailand; 5 HPV & EBV and Carcinogenesis Research Group, Khon Kaen University, Khon Kaen, Thailand

**Keywords:** Human papillomavirus, Herpes simplex virus, Epstein-Barr virus, Co-infection, Asymptomatic MSM, Anatomical site

## Abstract

**Background:**

Human papillomavirus (HPV), Epstein-Barr virus (EBV) and herpes simplex virus **(**HSV) cause sexually transmitted diseases (STDs) that are frequently found in men who have sex with men (MSM) with human immunodeficiency viral (HIV) infection.

**Methods:**

This study investigated the prevalence of infection and anatomical site distribution of these viruses in asymptomatic MSM. DNA, extracted from cells collected from the anorectum, oropharynx and urethra of 346 participants, was investigated for the presence of EBV, HPV and HSV using real-time PCR. Demographic data from the participants were analyzed.

**Results:**

All three viruses were found in all sampled sites. EBV was the commonest virus, being detected in the anorectum (47.7% of participants), oropharynx (50.6%) and urethra (45.6%). HPV and HSV were found in 43.9% and 2.9% of anorectum samples, 13.8% and 3.8% of oropharynx samples and 25.7% and 2% of urethra samples, respectively. HPV infection of the anorectum was significantly associated with age groups 21–30 (odds = 3.043, 95% CI = 1.643–5.638 and *P* = 0.001) and 46–60 years (odds = 2.679, 95% CI = 1.406–5.101 and *P* = 0.03). EBV infection of the urethra was significantly correlated with age group 21–30 years (odds = 1.790, 95% CI = 1.010–3.173 and *P* = 0.046). EBV/HPV co-infection of the anorectum (odds = 3.211, 95% CI = 1.271–8.110, *P* = 0.014) and urethra (odds = 2.816, 95% CI = 1.024–7.740, *P* = 0.045) was also associated with this age group. Among HIV-positive MSM, there was a significant association between age-group (odds = 21.000, 95% CI = 1.777–248.103, *P* = 0.016) in HPV infection of the anorectum. A failure to use condoms was significantly associated with HPV infection of the anorectum (odds = 4.095, 95% CI = 1.404–11.943, *P* = 0.010) and urethra (odds = 7.187, 95% CI = 1.385–37.306, *P* = 0.019). Similarly, lack of condom use was significantly associated with EBV infection of the urethra (odds = 7.368, 95% CI = 1.580–34.371, *P* = 0.011).

**Conclusion:**

These results indicate that asymptomatic MSM in Northeast Thailand form a potential reservoir for transmission of STDs, and in particular for these viruses.

**Electronic supplementary material:**

The online version of this article (10.1186/s12879-018-3406-0) contains supplementary material, which is available to authorized users.

## Background

Men who have sex with men (MSM) are at higher risk of sexually transmitted diseases (STDs) than other groups. Incidence has been rising due to the practice of various sexual acts including penile–anal, oral–anal, and/or penile–oral contact [1, 2]. HPV is the most common sexually transmitted viral infection and its prevalence is increasing [3]. Human papillomavirus (HPV) is a causative agent of genital warts [4] and the main risk factor for anal cancer in MSM [5]. The incidence of anal cancer is highest in HIV-infected MSM and is increasing annually. HPV is also associated with oropharyngeal and penile cancers, but at lower prevalence than anal cancer [6]. Detection of HPV infection in asymptomatic MSM can be used to monitor and follow-up HPV-persistent infection for HPV-related cancer intervention.

Other viruses are also found in MSM. Epstein-Barr virus (EBV) is one of the most common human viruses found in B-cells and epithelial cells of healthy persons [7]. The presence of EBV in sites such as the anus, oropharynx and urethra can be due not only to intimate contact but also to the movement of EBV-infected B-cells. Most people are infected in childhood and do not develop symptoms, or have very minor symptoms such as a mild infectious mononucleosis syndrome [8]. EBV has been frequently found in the non-genital and genital mucosa, ulcers and urethral discharges and associated with various malignancies including Burkitt’s lymphoma, Hodgkin’s disease, non-Hodgkin’s lymphoma, nasopharyngeal carcinoma, breast cancer, gastric cancer, etc. [9]. It seems likely to be a co-factor in HPV-associated cancers such as anal and penile cancers [10, 11]. Moreover, EBV infection is associated with HPV integration into the host genome, which is a relevant process in cervical cancer progression [12]. In contrast, EBV is more frequently found than HPV in oropharyngeal cancer [13, 14]. Interestingly, the prevalence of EBV in isolated B-cells of MSM is significantly higher than in heterosexual men [15]. MSM appear to be at more risk of EBV infection. Although EBV causes various types of disease, including cancer, its co-prevalence with HPV among asymptomatic MSM at various anatomical sites has been little studied.

Herpes simplex virus (HSV) is one of the commonest sexually transmitted viral infections worldwide. The usual sites of HSV infection are skin and mucosal membranes. Primary infection sites of HSV-1 and HSV-2 are the oropharynx and genital tract, respectively. Infection is often asymptomatic. Even though HSV-2 is predominantly spread via the genital route (in contrast to HSV-1) and its seroprevalence is higher in HIV-positive (> 80%) than HIV-negative MSM [16], HSV-1 is causing an increasing proportion of anogenital herpes worldwide [17, 18]. Anogenital HSV-1 is more common in MSM than heterosexual individuals [19]. Interestingly, HSV infection is associated with increased viral load of HIV in infected MSM [20]. Interestingly, co-infection of HSV and HPV16 in patients with head and neck carcinomas (HNSCC) has the worst disease outcome [21]. In addition, HSV-1 infection may modulate the radiation resistance of HPV16-positive HNSCC cells by improving cell survival after irradiation [22]. Therefore, HSV can be a co-factor of HPV-associated carcinogenesis and may be a main reservoir in MSM. We, therefore, investigated HSV in MSM.

To explore the prevalence and anatomical site distribution of HPV, EBV and HSV infecting asymptomatic MSM in Northeast Thailand, real-time polymerase chain reaction (RT-PCR) was used to detect these viruses from 346 participants at anatomical sites including the oropharynx, urethra and anorectum.

## Methods

### Specimen and data collection

In total, 358 asymptomatic MSM were enrolled under a cross-sectional study project title of “Factors associated to *Neisseria gonorrhea* infection by anatomic distributions among men who have sex with men, and multidrug resistant patterns of *Neisseria gonorrhea*” at M-Reach STDs clinic in Chatapadung Contracting Medical Unit, and the ARV Clinic, Khon Kaen Hospital, Khon Kaen Province, Thailand from September 2014 to July 2015. Prevalence of *Neisseria gonorrhea* in urethra was published [23, 24], but not in oropharynx and anorectum. Cell samples from anorectum, oropharynx and urethra were collected using sterile Dacron swabs (Puritan, Hardwood Products, Guilford, USA). These swabs were immediately transferred into 2 ml of 10% formalin in normal saline solution and transported to laboratory on ice within 4 h. Three-hundred and forty-six asymptomatic MSM were included whereas 12 MSM were excluded because samples were not collected from all three anatomical sites. Participants provided basic demographic data and information concerning their sexual behavior, including number of sexual partners in the preceding 3 months, condom usage and HIV status. This was done by means of a self-reported questionnaire and data were recorded in an anonymous electronic file. The ethical approval for this study was obtained from Khon Kaen University Ethics Committee in Human Research, No. HE591377.

### DNA extraction

Cells from swab samples were pelleted and washed with phosphate buffered saline by centrifugation at 2000 rpm for 5 min. Cells were lysed using lysis buffer (10 mM Tris HCl, 0.1 mM EDTA pH 7.5, 1% SDS and 0.5 M NaCl) supplemented with 50 mg/ml of proteinase K and then incubated at 60 °C for 30 min. Protein was precipitated by addition of protein precipitation buffer (5 M potassium acetate, 11.5 ml of glacial acetic acid and 28.5 ml of distill water, pH 5.5), and then removed by centrifugation at 13,500 rpm for 5 min at 4 °C. DNA was precipitated with an equal volume of isopropanol and collected by centrifugation at 13,500 rpm for 5 min at 25 °C and washed with 70% ethanol. Finally, the DNA pellet was dried at 37 °C for 15–30 min and then resuspended in distilled water. The quality of DNA was checked by amplifying the *GAPDH* gene using specific primers (GAPDH forward: 5′-TCATCAGCAATGCCTCCTGCA-3′ and reverse: TGGGTGGCAGTGATGGCA-3′ by RT-PCR. Quantity of DNA was assessed using the NanoDrop™ (Thermo Scientific) [25].

### Detection of HPV, HSV and EBV infection by RT-PCR

HPV infection was investigated using GP5+/GP6+ primers (forward: 5′-TTTGTTACTGTGGTAGATACTAC-3′ and reverse: 5′-GAAAAATAAACTGTAAATCATATTC-3′) by RT-PCR [26] to amplify a 141 bp portion of the L1 viral capsid gene. The reaction mixture had a final volume of 20 μl containing 1× SsoAdvancedTM Universal SYBR® Green Supermix (Bio-Rad, Hercules, CA, USA), 0.2 μM of forward primer, 0.2 μM of reverse primer and DNA template. Thermocycling conditions were a denaturation step of 5 min at 95 °C followed by 45 cycles of 95 °C for 10 s and 42 °C for 30 s in an Applied Biosystems 7500 Fast real-time PCR Instrument (Applied Biosystems, Foster City, CA, USA). DNA from SiHa cells (an HPV16-positive cell line) was used as the positive control for HPV DNA detection.

HSV infection was detected using specific primers: HSV DNA polymerase forward: 5′- GTGTTGTGCCGCGGTCTCAC-3′ and reverse: 5′-GGTGAACGTCTTTTCGAACTC-3′. EBV was detected using EBV DNA polymerase forward: 5′- GGAGAAGGTCTTCTCGGCCTC-3′ and reverse: 5′-TTCAGAGAGCGAGACCCTGC-3′ [27, 28]. The reaction mixture had a final volume of 20 μl containing 1× SsoAdvancedTM Universal SYBR® Green Supermix (Bio-Rad, Hercules, CA, USA), 0.2 μM of forward primer, 0.2 μM of reverse primer and DNA template. The reaction was performed in an Applied Biosystems 7500 Fast real-time PCR Instrument (ABi). Cycling conditions were; initial 3 min at 95 °C followed by 40 cycles of 95 °C for 10 s, 64 °C for 10 s and 72 °C for 30 s. DNA from HSV-1 kos particles and P3HR1 cells (an EBV-positive cell line) was used as the positive control for HSV DNA and EBV DNA detection, respectively.

### HPV genotyping

HPV L1 gene fragments in HPV-positive samples were amplified using GP5/ GP6+ primers labeled with biotin and genotyped by reverse line blot hybridization (RLBH) [29]. Biodyne C blotting membrane (Pall Life Science, Ann Arbor, MI, USA) was activated in 16% (*w*/*v*) 1-ethy-3-(3-dimethylaminopropyl) carbodimide (EDAC) solution (Sigma-Aldrich, St. Louis, MO, USA) at room temperature for 10 min, rinsed with distilled water and placed on a mini blotter. Thirty-seven HPV type-specific 5′-amino linked oligonucleotide probes, including 13 high-risk HPV types (16, 18, 31, 33, 35, 39, 45, 51, 52, 56, 58, 59 and 68); 12 low-risk HPV types (6, 11, 26, 40, 42, 43, 44, 53, 54, 61, 72 and 73); and other HPV types (34, 55, 57, 66, 70, 82MM4, 83MM7, 84MM8, 82IS39, CP6108, 71CP8061 and 81CP8304), were dropped onto the Biodyne C membrane through the wells of the mini blotter in parallel lines. Subsequently, biotin-labeled PCR products were added into the channels of the mini blotter perpendicular to the oligonucleotide probe lines, then hybridized and incubated with streptavidin-peroxidase-conjugate. The HPV types present were detected using chemiluminescence.

### Statistical analysis

Bivariate analysis (for comparisons of proportions) was used to investigate the association between EBV, HPV and/or HSV infection and age, number of sex partners, condom usage and HIV status of participants using SPSS software (SPSS Inc., Chicago, IL, USA). Any *P*–value < 0.05 was considered statistically significant.

## Results

### Patient characteristics

Table 1 shows demographic data and sexual behavior characteristics of 346 asymptomatic MSM participants. The age range was 18–60 years, with the mode being the 21–30-year-old group. Most participants had only one sexual partner, or no partners, in the previous 3 months and always used condoms. A total of 234 asymptomatic MSM self-revealed HIV status.

**Table 1:** Clinical data of MSM (*n* = 346)

Clinical finding	n = 346
Age (years)	
Minimum = 18	60
Maximum = 60	1
Age-range groups	
18–20	76
21–30	139
31–45	106
46–60	25
Number of partners within 3 months	
None	129
1–2	166
> 2	51
Condom usage	
Always	223
Sometimes	64
Never	59
HIV status	
Negative	124
Positive	110
Unknown	112

### Prevalence of EBV, HPV or HSV infecting MSM and anatomical site distribution

**Fig. 1:**
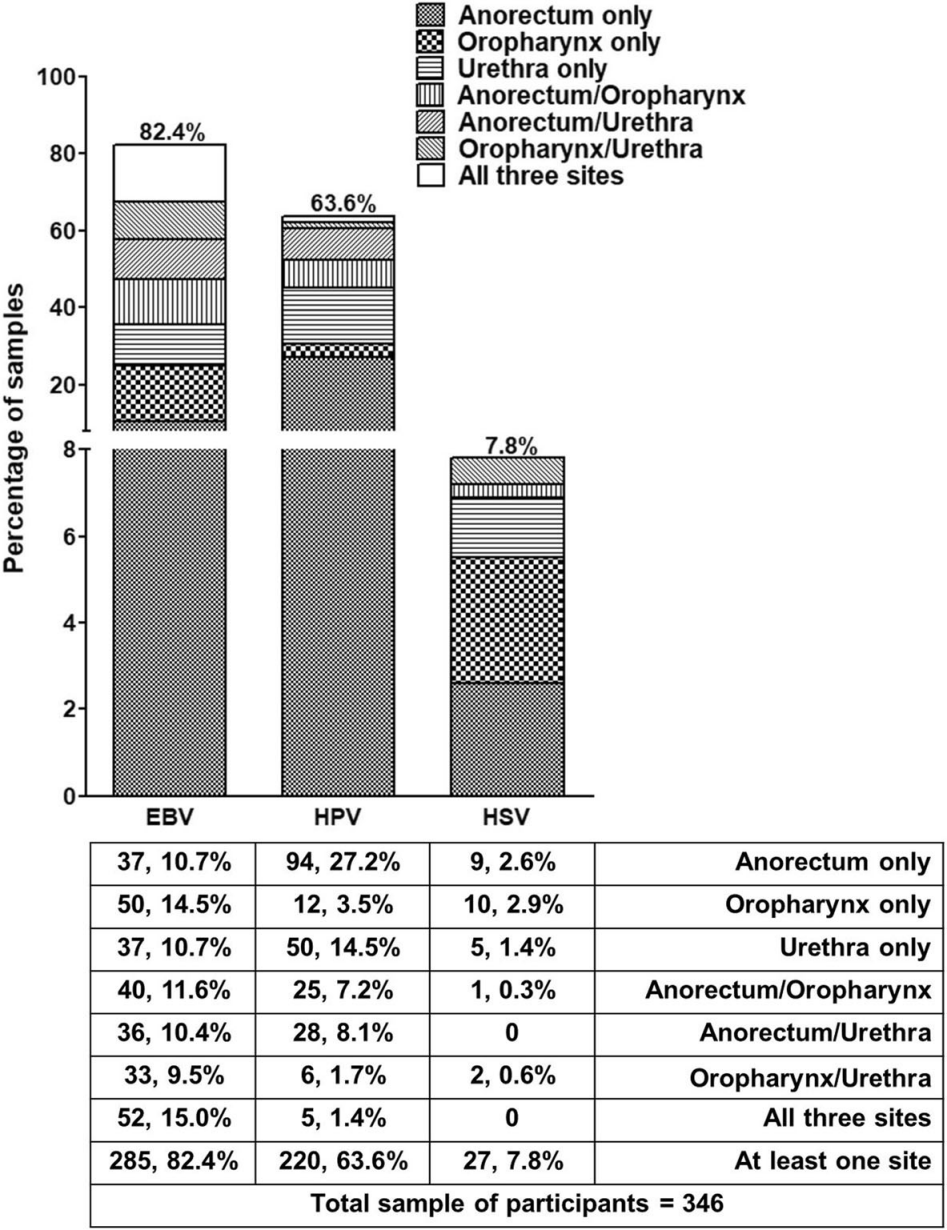
Prevalence of EBV, HPV and HSV in the anorectum, oropharynx and urethra in 346 asymptomatic MSM (n, %)

Swab samples were obtained from each anatomical site (anorectum, oropharynx and urethra) of 346 asymptomatic MSM participants. HPV, EBV and HSV infection were detected in all three anatomical sites (Fig. 1). EBV infection was common at all three sites, with approximately 50% of samples returning a positive result from each (Fig. 1). The anorectum was the site where HPV infection was most common (43.9%), followed by the urethra (25.7%) and oropharynx (13.9%). HSV was the least prevalent (Fig. 1).

**Fig. 2:**
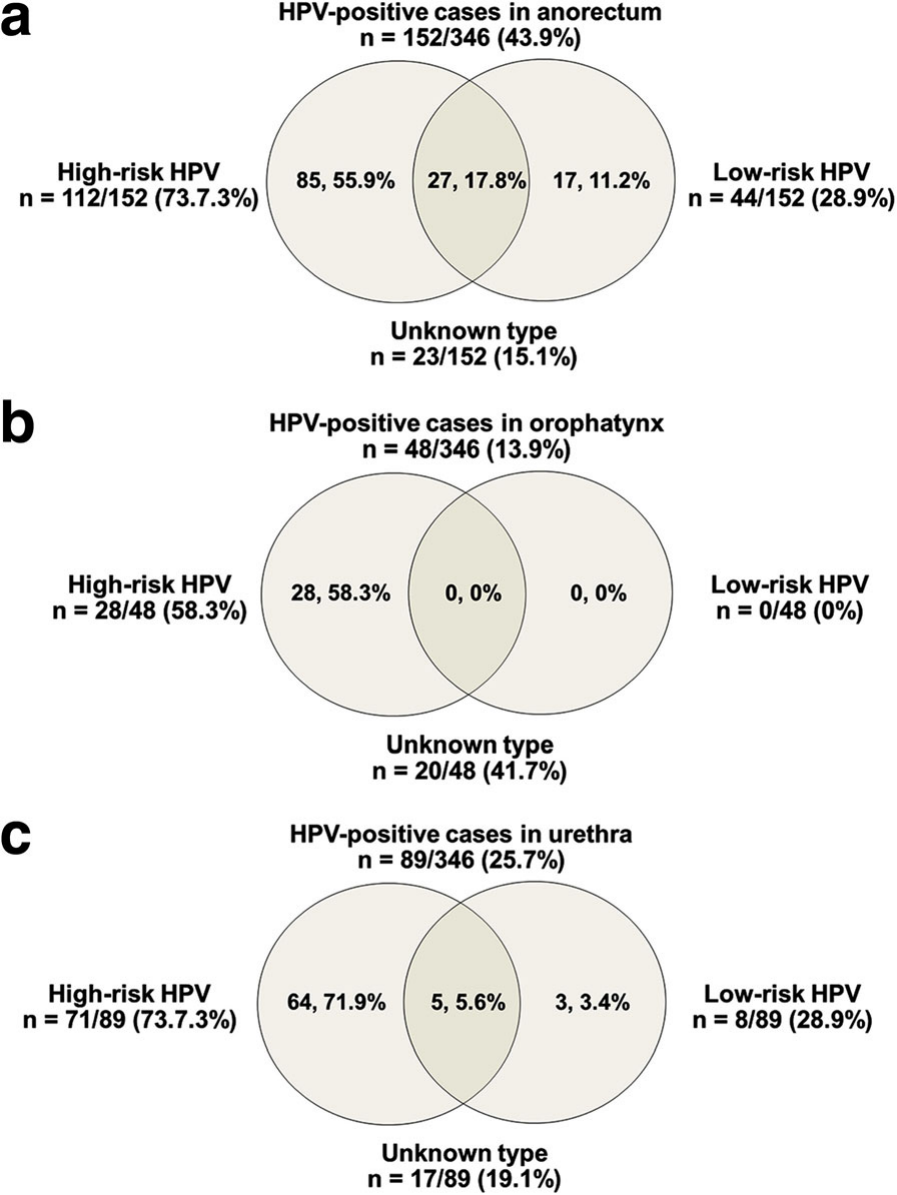
Site distribution of HPV risk types in HPV-positive cases. **a** HPV-positive cases in anorectum, **b** HPV-positive cases in oropharynx and **c** HPV-positive cases in urethra

The prevalence of high-risk HPV types were higher than of low-risk HPV in all anatomical sites (Fig. 2). Double/multiple infections of high and low-risk types were particularly frequent in the anorectum (Fig. 2a), followed by the urethra (Fig.  2c), but a combination of risk types did not occur in the oropharynx (Fig.  2b). The anorectal site seems to be the main reservoir of infection of HPV high- and low-risk types in MSM.

The highest prevalence of double or multiple infections of any type of HPV was in the anorectum (67/152, 44.1%) followed by the urethra (21/89, 23.6%) and oropharynx (3/48, 6.3%). Infections with double and multiple HPV types were more common than with single HPV types in the anorectum (44.1% vs. 40.1%, with HPV types 18 and 58 being the most common combination), but not in the oropharynx (6.3% vs. 52.1%, with HPV types 39 and 58 being the most common combination) or urethra (23.6% vs. 57.3%, with HPV types 16 and 18 being the most common combination) (Additional file 1: Table S1). HPV 18 was mostly found in the anorectum (42/152, 27.6% followed by HPV 16 and HPV 58 in similar proportions at 25%) and in the urethra (40/89, 44.9% followed by HPV 16 and HPV 58 in 21.3% and 19.1% respectively). HPV 58 was most frequently detected in the oropharynx (18/48, 37.5% followed by HPV types 39, 18 and 53 at 8.3%, 6.3% and 6.3% respectively).

### Factors associated with prevalence of HPV, EBV and HSV in the anorectum, oropharynx and urethra among 346 asymptomatic MSM

There were often significant differences between age groups in prevalence and anatomical sites, as shown in Table 2. EBV infection in the urethra (but not the anorectum or oropharynx) was significantly associated with the 21–30 years-old group (odds = 1.790, 95% CI = 1.010–3.173 and *P* = 0.046). HPV infection in the anorectum was significantly associated with the 21–30 years-old group (odds = 3.043, 95% CI = 1.643–5.638 and *P* = 0.001) and also the 46–60 years-old group (odds = 2.679, 95% CI = 1.406–5.101 and *P* = 0.03). HSV infection in the oropharynx was mostly found in the 46–60 years-old group. EBV infection in the oropharynx and urethra was significantly higher among HIV-positive MSM than among HIV-negative MSM (odds = 2.125, 95% CI = 1.257–3.594 and *P* = 0.005 and odds = 2.536, 95% CI = 1.496–4.298 and *P* = 0.001, respectively). Likewise, HPV infection in the anorectum was significantly associated with HIV-infected MSM (odds = 1.935, 95% CI = 1.150–3.257 and *P* = 0.013). In contrast, the incidence of HSV did not differ according to HIV status. This result suggested that HIV-infected MSM might act as reservoirs for transmission of EBV and HPV.

**Table 2:** Prevalence of EBV, HPV or HSV in age range, number of partners within 3 months, condom usage and HIV status in the anorectum, oropharynx and urethra among 346 asymptomatic MSM

Factors	Anorectum, n (%)			Oropharynx, n (%)			Urethra, n (%)		
	EBV	HPV	HSV	EBV	HPV	HSV	EBV	HPV	HSV
Age range (years)									
18–20 (*n* = 76)	35 (46.1)	19 (25.0)	3 (3.9)	41 (53.9)	7 (9.2)	0	28 (36.8)	18 (23.7)	2 (2.6)
21–30 (*n* = 139)	68 (48.9)	70 (50.4)*	3 (2.2)	77 (55.4)	25 (18.0)	3 (2.2)	71 (51.1)*	42 (30.2)	2(1.4)
31–45 (*n* = 106)	51 (48.1)	50 (47.2)	3 (2.8)	44 (41.5)	10 (9.4)	7 (6.6)	48 (45.3)	23 (21.7)	3 (2.8)
46–60 (*n* = 25)	11 (44.0)	13 (52.0) *	1 (4.0)	13 (52.0)	6 (24.0)	3 (12.0)	11 (44.0)	6 (24.0)	0
Number of partners within 3 months									
None (*n* = 129)	56 (43.4)	57 (44.2)	2 (1.6)	74 (57.4)	17 (13.2)	3 (2.3)	66 (51.2)	30 (23.3)	5 (3.9)
1–2 (*n* = 166)	83 (50.0)	78 (47.0)	5 (3.0)	78 (47.0)	28 (16.9)	8 (4.8)	72 (43.4)	45 (27.1)	0
> 2 (*n* = 51)	26 (51.0)	17 (33.3)	3 (5.9)	23 (45.1)	3 (5.9)	2 (3.9)	20 (39.2)	14 (27.5)	2 (3.9)
Condom usage									
Always (*n* = 223)	10 (47.1)	98 (43.9)	7 (3.1)	111 (49.8)	30 (13.5)	7 (3.1)	97 (43.5)	54 (24.2)	5 (2.2)
Sometimes (*n* = 63)	36 (56.3)	23 (35.9)	2 (3.1)	33 (51.6)	6 (9.4)	3 (4.7)	28 (43.8)	21 (32.8)	1 (1.6)
Never (*n* = 60)	24 (40.7)	31 (52.5)	1 (1.7)	31 (52.5)	12 (20.3)	3 (5.1)	33 (55.9)	14 (23.7)	1 (1.7)
HIV status									
Negative (*n* = 124)	60 (48.4)	53 (42.7)	6 (4.8)	56 (45.2)	21 (16.9)	6 (4.8)	45 (36.3)	30 (24.2)	2 (1.6)
Positive (*n* = 110)	53 (48.2)	65 (59.1)*	4 (3.6)	70 (63.6)*	16 (14.5)	5 (4.5)	65 (59.1)*	32 (29.1)	3 (2.7)
Unknown (*n* = 112)	52 (46.4)	34 (30.4)	0	49 (43.8)	11(9.8)	2 (1.8)	48 (42.9)	27 (24.1)	2 (1.8)

* denotes significant difference at *P* < 0.05 by multivariate analysis

Table 3 shows the correlation between HPV and EBV infection in HIV-infected and uninfected MSM with the associated factors. HPV infection in the anorectum in HIV-positive MSM was significantly associated with increasing age. For the 31–45 years-old group, odds = 10.500, 95% CI = 1.177–93.697, *P* = 0.035; and for the 46–60 years-old group, odds = 21.000, 95% CI = 1.777–248.103, *P* = 0.016. Co-infection of HIV with HPV-infected anorectum (odds = 4.095, 95% CI = 1.404–11.943 and *P* = 0.010), urethra (odds = 7.187, 95% CI = 1.385–37.306, *P* = 0.019) or EBV-infected urethra (odds = 7.368, 95% CI = 1.580–34.371, *P* = 0.011) was significantly associated with an absence of condom usage. HPV infection in the oropharynx wasn’t associated with any demographic factors (data not shown). In addition, no association was found between demographic factors and EBV infection in the anorectum and oropharynx among HIV-infected and uninfected MSM (data not shown). This result demonstrated that lack of condom usage was an important factor for HPV infection in the anorectum and urethra as well as EBV infection in the urethra among HIV-positive MSM.

**Table 3:** The association of demographic factors with HPV infection in the anorectum and urethra and with EBV infection in the urethra among HIV-infected MSM

Factors	HPV-infected anorectum		HPV-infected urethra		EBV-infected urethra	
	HIV +	Odds, 95%CI, *P*-value	HIV +	Odds, 95%CI, *P*-value	HIV +	Odds, 95%CI, *P*-value
Age range (years)						
18–20 (n = 76))	1 (12.5)	Reference	0 (0)	–	3 (42.9)	Reference
21–30 (n = 139)	31 (53.5)	8.037, 0.929–69.542, 0.058	18 (53.0)	Reference	35 (61.4)	2.121, 0.433–10.392, 0.354
31–45 (n = 106)	24 (60.0)	10.500, 1.177–93.697, 0.035	9 (50.0)	0.889, 0.283–2.789, 0.840	22 (59.5)	1.956, 0.381–10.026, 0.421
46–60 (n = 25)	9 (75.0)	21.000, 1.777–248.103, 0.016	5 (83.3)	4.444, 0.468–42.175, 0.194	5 (55.6)	1.667, 0.227–12.221, 0.615
No. of partners within 3 mouths						
None (n = 129)	30 (66.7)	Reference	13 (68.4)	Reference	28 (62.2)	Reference
> 1 (*n* = 217)	35 (48.0)	0.461, 0.213–1.000, 0.050	19 (44.2)	0.365, 0.117–1.142, 0.083	37 (56.9)	0.802, 0.369–1.745, 0.579
Condom usage						
Always (n = 223)	40 (50.6)	Reference	16 (41.0)	Reference	38 (52.0)	Reference
Sometimes (n = 63)	4 (30.8)	0.433, 0.123–1.524, 0.193	6 (54.5)	1.725, 0.448–6.637, 0.428	11 (57.9)	1.266, 0.457–3.512, 0.650
Never (n = 60)	21 (80.8)	4.095, 1.404–11.943, 0.010	10 (55.9)	7.187, 1.385–37.306, 0.019	16 (88.0)	7.368, 1.580–34.371, 0.011

### Co-infection with EBV, HPV and/or HSV in three anatomical sites

**Fig. 3:**
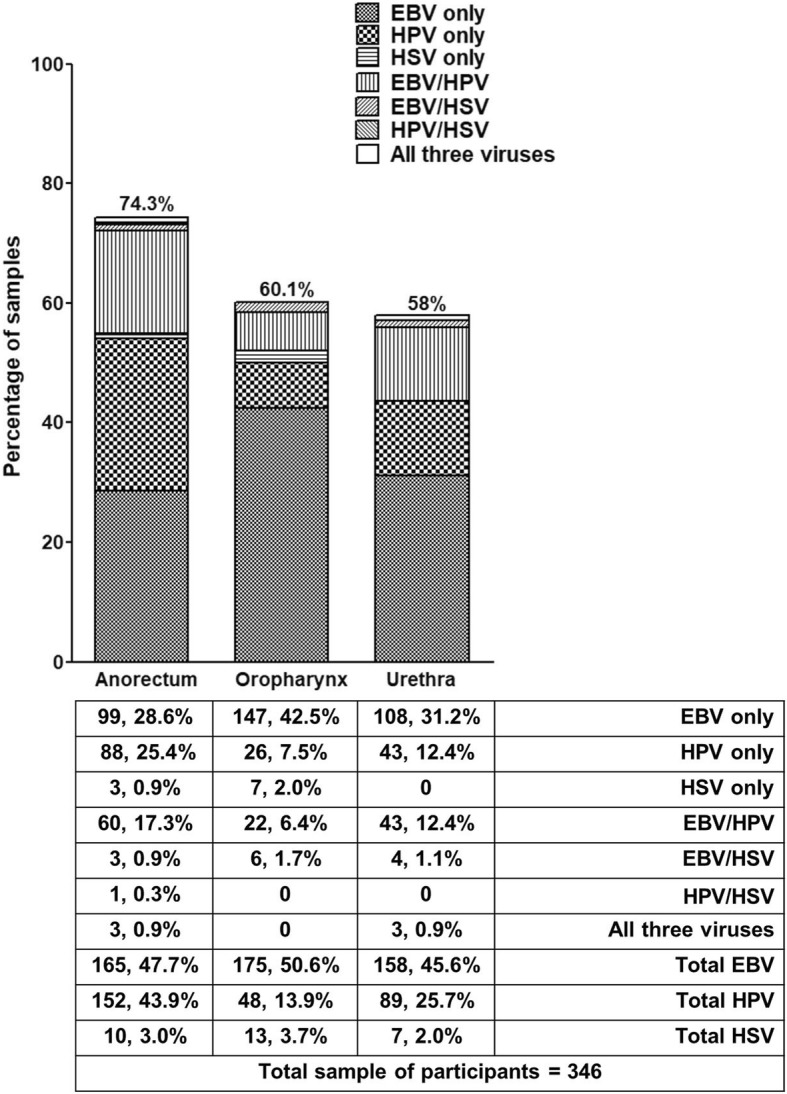
Prevalence and anatomical site distribution of EBV, HPV and EBV in 346 asymptomatic MSM (n, %)

Co-infection with EBV and HPV was common, especially in the anorectum (17.3%) (Fig. 3). Reflecting its low prevalence generally, co-infection of HSV with either of the other two viruses was uncommon (Fig. 3). All three viruses were found in 0.9% (3/346) of MSM in the anorectum or urethra but not in the oropharynx (Fig. 3). These results demonstrate that co-infection of EBV and HPV common among northeast Thai MSM at all three anatomical sites.

### The association of demographic factors and co-infection of EBV, HPV and HSV in anorectal, oropharyngeal and urethal sites among asymptomatic MSM

Co-infection of HPV with EBV or HSV in all three anatomical sites has not been reported previously. We investigated the association of EBV, HPV and/or HSV co-infection in anorectal, oropharyngeal or urethral sites with demographic factors. Interestingly, co-infection with EBV and HPV at both the anorectum and urethra was significantly associated with the 21–30 years-old group (odds = 3.211, 95% CI = 1.271–8.110, *P* = 0.014 and odds = 2.816, 95% CI = 1.024–7.740, *P* = 0.045 respectively), as shown in Additional file 2: Tables S2 and Additional file 3: Tables S3. In addition, EBV and HPV co-infection in the anorectum and urethra was most found frequently in MSM with current or recent partners and in HIV-positive MSM. Meanwhile, EBV and HPV co-infection at the oropharyngeal site was most frequent in the 45–60 years-old group, as well as in HIV-positive MSM (Additional file 4: Table S4). Co-infections of EBV with HSV, and of HPV with HSV, and of all three viruses together, were not associated with any demographic factors in any anatomical sites (Additional file 2: Tables S2, Additional file 3: Table S3, and Additional file 4: Tables S4). These findings show that co-infection of EBV with HPV was predominately found in the 21–30 years-old group in the anorectum and urethra but not in the oropharynx.

## Discussion

In this study, we addressed the prevalence of HPV, EBV and HSV as single and co-infections in different anatomical sites (anorectum, oropharynx and urethra) of 346 MSM in Northeast Thailand. Demographic information was collected from participants, and especially HIV status. Several previous reports exist about the prevalence of EBV in the same anatomical sites of MSM from other countries. EBV infection was found in the anorectum of 29.6% and 32% of HIV-positive German and Swedish MSM, respectively [30, 31]. Oropharyngeal shedding of EBV was detected in 49–88.8% and 16–56% of HIV-seropositive and seronegative MSM, respectively [32, 33]. In the urethral site, EBV was found in 30.7% of asymptomatic American MSM; notably, EBV prevalence (72.7%) was significantly associated with HIV shedding in semen [34], corresponding to our finding as shown in Table 2. EBV infection of the urethra ranged from 28 to 30.7% among American MSM [34, 35]. Meanwhile, EBV infection of the urethra of American and Spanish men was ranged from 0.4–45% [36]. Most of these published articles demonstrated that EBV had the highest prevalence in the oropharynx, in contrast to the anorectum and urethra, corresponding to our results.

Many studies have demonstrated a high prevalence of HPV infection in the anorectum, ranging from 34.8 to 65.3% [37–39], and was significantly associated with HIV-infected MSM [37, 38], concordant with our results. Prevalence of HPV infection were lower in the oropharynx and urethra (9.6–13.0% and 10.2–16.3%, respectively) [37, 40–42]. As is the case for the anorectum, high prevalence of HPV infection at the oropharynx and urethra were significantly associated with HIV-positive MSM [41, 42].

In Peruvian MSM, the five most common HPV genotypes from the anorectum were 53, 6, 16, 58 and 54 [43]. Similarly, HPV53 was the most frequently found genotype in anorectal samples from HIV-seropositive French MSM [44], but HPV16 was the most frequently found at this site in Chinese MSM where it was not associated with HIV status. However, HPV6, 18, 31, 39, 45 and 66 were significantly associated with HIV infection in China [39]. HPV16 was the most common genotype found in the anorectum of Italian MSM [45]. However, HPV18 was the most frequently found genotype in the anorectum and urethra of Northeast Thai MSM. In the urethral site, HPV58 was the most common in Italian MSM. HPV16 and HPV58 were the most common infections of the oropharynx of MSM in The Netherlands and Italy respectively [40, 42]. Concordantly, our study has also demonstrated the highest proportion of HPV58 in the oropharynx. Although HPV16 was the most common genotype detected in these three anatomical sites among South African and Dutch MSM [42, 46], there appears to be variation by region and nationality. Co-infections with two or more HPV genotypes are significantly more frequent than single infections [43, 44], consistent with our findings and particularly in the anorectum. Unsurprisingly, high-risk HPV has been more frequently detected than low-risk genotypes among MSM worldwide [39, 44].

Most studies of HSV infection in MSM have reported only seroprevalence (82.5–90%) [47, 48]. However, a few studies have reported prevalence of HSV DNA in the anorectum (ranging from 7.0 to 16.9%), oropharynx (0–7.2%) and urethra (2.3–49.5%) among MSM [11, 34, 49–51]. Consistent with previous studies, we found very low prevalence of HSV in all three sites (Fig. 1).

Our report is the first to find that EBV infection is present in anorectal, oropharyngeal and urethral sites of northeast Thai MSM at higher prevalence than are HPV and HSV. A previous study found high-risk HPV at higher prevalence (90.8%) in the anorectal canal than low-risk-HPV (73.8%), HSV-1 (7.7%), HSV-2 (16.9%) and EBV (7.7%) among HIV-positive Brazilian men [11]. This agreed with a Swedish study of anal cell samples, which found higher HPV infection (76%) among HIV-infected and uninfected MSM than EBV (18.7%) and HSV (9.4%) [30].

HPV was also the most common virus found in anal swabs (44%), followed by semen (7.1%) and pharyngeal swabs (3.8%) in American MSM [35], consistent with our finding that HPV infection was more frequent in the anorectum than in the urethra and oropharynx.

It is well known that HIV-infected MSM have an increased risk of sexually transmitted infections (STIs) and STDs [52]. In addition, HIV infection not only increases susceptibility to persistent HPV but also increases the risk of acquisition of new HPV infections [53]. Similarity, EBV shedding was significantly associated with persistent HPV infection among HIV-infected MSM in the USA [35]. Our finding demonstrated that the presence of HPV in the anorectum was significantly associated with HIV infection in MSM (Table 2). We also found EBV infection of the oropharynx and urethra to be significantly associated with HIV-positive MSM (Table 2). Previous studies suggested that prevalence of EBV, HPV and HSV infection were associated with HIV-infected MSM but not HIV-uninfected MSM [30].

A few studies have reported co-infection of EBV, HPV and/or HSV in asymptomatic MSM. For example, high-risk HPV/HSV-2 co-infections were present in the anorectum of 55% of Brazilian MSM. Corresponding values for other combinations in that study were 27% (high-risk HPV/low-risk HPV/EBV), 9% (high-risk HPV/low-risk HPV/HSV2/EBV) and 9% (high-risk HPV/low-risk HPV/HSV-1/HSV-2/EBV) [11]. HPV and HSV co-infections were found in 4.3% of oral samples from Finnish males [50]. We have demonstrated that co-infection of EBV with HPV was the most common in the anorectum (17.3%), oropharynx (6.4%) and urethra (12.4%) and was significantly associated with the 21–30 years-old group (Fig. 3 and Additional file 2: Tables S2, Additional file 3: Table S3, and Additional file 4: Tables S4).

Different results in prevalence in anatomical sites of three viruses and in the association with any risk factors among various countries may depend on the technique used, site and equipment of sample collection, sample size, sexual behavior, nationality, geography, and particularly questionnaire pattern, etc. (such as a self-report or community based). The strength of our study is that 1) we used swab sample in urethra because this can increase sensitivity of *Neisseria gonorrhea* detection compared with urine sample [24] whereas many previous studies performed EBV, HPV and HSV detection in MSM semen [35, 36]; additionally the cell samples can indicate true infection at each anatomical site instead of movement of EBV infected B cells to each sites; and 2) all participants recruited in our study are collected cell samples from all anatomical sites to compare each participants that can reduce the error of data. However, there was a limitation in our study including self-reporting MSM that may provide invalid answers such as HIV status [54]. This limitation may cause an inaccurate data analysis of demographic information.

Here, we also investigated HIV status among EBV, HPV or HSV-positive MSM. We found that HPV infection of the anorectum increased with age among HIV-infected MSM (Table 3). In addition, failure to use condoms by HIV-positive MSM was significantly associated with HPV infection of the anorectum and urethra as well as with EBV infection of the urethra (Table 3).

## Conclusions

EBV and HPV were detected in asymptomatic MSM in Northeast Thailand and were found more frequently than HSV in all three anatomical sites (oropharynx, anorectum and urethra). Oncogenic high-risk HPV genotypes were highly prevalent at these three sites in this population. Therefore, molecular detection and HPV genotyping may be useful for identifying these viruses and facilitating interventions to limit their spread among MSM, especially HIV-infected MSM.

## Additional files

Additional file 1: **Table S1.** Single and double/multiple infections of different HPV types in the anorectum, oropharynx and urethra. (DOCX 15 kb) (DOCX 16 kb)

Additional file 2: **Table S2.** The association of demographic factors and co-infection of EBV, HPV and/or HSV in the anorectal site. (DOCX 16 kb) (DOC 44 kb)

Additional file 3: **Table S3.** The association of demographic factors and co-infection of EBV, HPV and/or HSV in the urethral site. (DOCX 16 kb) (DOC 43 kb)

Additional file 4: **Table S4.** The association of demographic factors and co-infection of EBV, HPV and/or HSV in the oropharyngeal site. (DOCX 16 kb) (DOC 45 kb)

## Abbreviations

EBV: Epstein-Barr virus
HIV: human immunodeficiency virus
HNSCC: head and neck carcinomas
HPV: human papillomavirus
HSV: herpes simplex virus
MSM: Men who have sex with men
STD: sexually transmitted disease
STI: sexually transmitted infections
