# Supplementary material for: Prevalence and anatomical sites of human papillomavirus, Epstein-Barr virus and herpes simplex virus infections in men who have sex with men, Khon Kaen, Thailand
Source: BMC Infect Dis. 2018 Oct 11;18:509. doi: 10.1186/s12879-018-3406-0 (PMC6180447; doi:10.1186/s12879-018-3406-0)
Supplement: Supplementary file 1 — Table S1. Single and double/multiple infections of different HPV types in the anorectum, oropharynx and urethra. (DOCX 15 kb) (DOCX 16 kb) [file 12879_2018_3406_MOESM1_ESM.docx]

**Supplementary Table 1** Single and double/multiple infections of different HPV types in the anorectum, oropharynx and urethra.

| HPV types | HPV in anorectal site, n = 152  n (%) |
| --- | --- |
| Double/multiple infections | 67 (44.1) |
| 18/58 | 6 (8.9) |
| 16/18 | 5 (7.5) |
| 16,58 | 5 (7.5) |
| Other double infections of high-risk and/or low-risk types | 28 (41.8) |
| Other multiple infections of high-risk and/or low-risk types | 23 (34.3) |
| Single infection | 61 (40.1) |
| HPV18 | 19 (31.2) |
| HPV16 | 11 (18.0) |
| HPV58 | 11 (18.0) |
| Other high-risk types (35, 39, 45b, 52, 56 or 66) | 11 (18.0) |
| Other low-risk types (6, 11, 42, 43, 61 or CP6108) | 9 (14.8) |
| Unknown | 24 (15.8) |
|  |  |
| HPV types | HPV in oropharyngeal site, n = 48  n (%) |
| Double/multiple infection | 3 (6.3) |
| 39/58 | 2 (66.7) |
| 52/58 | 1 (33.7) |
| Single infection | 25 (52.1) |
| 58 | 15 (60.0) |
| 13 | 3 (12.0) |
| 39 | 2 (8.0) |
| 52 | 2 (8.0) |
| Other high-risk types (16, 33 or 56) | 3 (12.0) |
| Other low-risk types | 0 (0) |
| Unknown | 20 (41.6) |
|  |  |
| HPV types | HPV in urethral site, n = 89  n (%) |
| Double/multiple infection | 21 (23.6) |
| 16/58 | 8 (38.1) |
| 18/58 | 3 (3.4) |
| Other double infections of high-risk and/or low-risk types (11/16, 56/58, 16/42, 18/39, 16/52, 16/58 and 18/42) | 8 (38.1) |
| Other multiple high-risk and/or low-risk types (11/18/58 and 16/18/42) | 2 (2.2) |
| Single infection | 51 (57.3) |
| 18 | 28 (54.9) |
| 58 | 10 (19.6) |
| 16 | 5 (9.8) |
| Other high-risk types (33, 39 and 52) | 5 (9.8) |
| Other low-risk types (11) | 3 (5.9) |
| Unknown | 17 (19.1) |
