# Supplementary material for: Prevalence and anatomical sites of human papillomavirus, Epstein-Barr virus and herpes simplex virus infections in men who have sex with men, Khon Kaen, Thailand
Source: BMC Infect Dis. 2018 Oct 11;18:509. doi: 10.1186/s12879-018-3406-0 (PMC6180447; doi:10.1186/s12879-018-3406-0)
Supplement: Supplementary file 2 — Table S2. The association of demographic factors and co-infection of EBV, HPV and/or HSV in the anorectal site. (DOCX 16 kb) (DOC 44 kb) [file 12879_2018_3406_MOESM2_ESM.doc]

**Supplementary Table 2 The association of demographic factors and co-infection of EBV, HPV and/or HSV in the anorectal site.**

| Factors | Anorectum | | | |
| --- | --- | --- | --- | --- |
|  | EBV/HPV | EBV/HSV | HPV/HSV | All 3 viruses |
| Age range (years) |  |  |  |  |
| 18-20, n = 76 | 6 (7.9) | 1 (1.3) | 0 | 0 |
| 21-30, n = 139 | 30 (21.6)* | 1 (0.7) | 0 | 1 (0.7) |
| 31-45, n = 106 | 20 (18.9) | 1 (0.9) | 1 (0.9) | 1 (0.9) |
| 45-60, n = 25 | 4 (16.0) | 0 | 0 | 1 (4.0) |
| Number of partners within 3 mouths |  |  |  |  |
| None, n = 129 | 20 (15.5) | 0 | 0 | 1 (0.8) |
| ≥ 1-2, n = 217 | 40 (18.4) | 3 (1.4) | 1 (0.4) | 2 (0.9) |
| Condom usage |  |  |  |  |
| Always, n =223 | 37 (16.6) | 3 (1.3) | 1 (0.4) | 0 |
| Sometimes, n = 64 | 12 (18.8) | 0 | 0 | 2 (3.1) |
| Never | 11 (18.6) | 0 | 0 | 1 (1.7) |
| HIV status |  |  |  |  |
| Negative, n = 124 | 21 (16.9) | 2 (1.6) | 0 | 1 (0.8) |
| Positive, n = 110 | 24 (21.8) | 1 (0.9) | 1 (0.9) | 2 (1.8) |
| Unknown, n = 112 | 15 (13.4) | 0 | 0 | 0 |
