# Supplementary material for: Prevalence and anatomical sites of human papillomavirus, Epstein-Barr virus and herpes simplex virus infections in men who have sex with men, Khon Kaen, Thailand
Source: BMC Infect Dis. 2018 Oct 11;18:509. doi: 10.1186/s12879-018-3406-0 (PMC6180447; doi:10.1186/s12879-018-3406-0)
Supplement: Supplementary file 4 — Table S4. The association of demographic factors and co-infection of EBV, HPV and/or HSV in the oropharyngeal site. (DOCX 16 kb) (DOC 45 kb) [file 12879_2018_3406_MOESM4_ESM.doc]

**Supplementary Table 4 The association of demographic factors and co-infection of EBV, HPV and/or HSV in the oropharyngeal site.**

| Factors | Oropharynx | | | |
| --- | --- | --- | --- | --- |
|  | EBV/HPV | EBV/HSV | HPV/HSV | All 3 viruses |
| Age range (years) |  |  |  |  |
| 18-20, n = 76 | 3 (3.9) | 0 | 0 | 0 |
| 21-30, n = 139 | 10 (7.2) | 1 (0.7) | 0 | 0 |
| 31-45, n = 106 | 7 (6.6) | 3 (2.8) | 0 | 0 |
| 45-60, n = 25 | 2 (8.0) | 2 (8.0) | 0 | 0 |
| Number of partners within 3 mouths |  |  |  |  |
| None, n = 129 | 10 (7.8) | 2 (1.6) | 0 | 0 |
| 1-2, n = 166 | 11 (6.6) | 4 (2.4) | 0 | 0 |
| > 2, n =51 | 1 (2.0) | 0 | 0 | 0 |
| Condom usage |  |  |  |  |
| Always, n =223 | 14 (6.3) | 3 (1.3) | 0 | 0 |
| Sometimes, n = 64 | 3 (4.7) | 1 (1.6) | 0 | 0 |
| Never | 5 (8.5) | 2 (3.4) | 0 | 0 |
| HIV status |  |  |  |  |
| Negative, n = 124 | 8 (6.5) | 2 (1.6) | 0 | 0 |
| Positive, n = 110 | 9 (8.2) | 4 (3.6) | 0 | 0 |
| Unknown, n = 112 | 5 (4.5) | 0 | 0 | 0 |
